# A qualitative study exploring hand hygiene practices in a neonatal unit in Blantyre, Malawi: implications for controlling healthcare-associated infections

**DOI:** 10.12688/wellcomeopenres.17793.2

**Published:** 2023-02-27

**Authors:** Helen Mangochi, Rachel Tolhurst, Victoria Simpson, Kondwani Kawaza, Kondwani Chidziwisano, Nicholas A. Feasey, Tracy Morse, Eleanor MacPherson

**Affiliations:** 1 Behaviour and Health Group, Malawi Liverpool Wellcome Clinical Programme, Blantyre, Malawi; 2 Liverpool School of Tropical Medicine, Liverpool, L3 5QA, UK; 3 Pediatrics, Queen Elizabeth Central Hospital, Blantyre, Malawi; 4 Kamuzu University of Health Sciences,, Blantyre, Malawi; 5 Centre for Water, Sanitation, Health and Appropriate Technology Development, Malawi University of Business and Applied Sciences, Blantyre, Malawi; 6 Department of Civil and Environmental Engineering, University of Strathclyde, Glasgow, UK

**Keywords:** Antimicrobial Resistance, Blood stream infections, Neonatal Sepsis, Infection Prevention and control practice, Water and Sanitation Hygiene (WASH)

## Abstract

**Background:** Neonatal sepsis is responsible for a considerable burden of morbidity and mortality in sub-Saharan African countries. Outcomes from neonatal sepsis are worsening due to increasing rates of antimicrobial resistance. Sub-optimal Infection Prevention and Control (IPC) practices of health care workers and caregivers are important drivers of infection transmission. The Chatinkha Neonatal Unit at Queen Elizabeth Central Hospital, Blantyre, Malawi has experienced multiple outbreaks of neonatal sepsis, associated with drug resistant Klebsiella pneumoniae. We aimed to understand the barriers to implementation of optimal IPC focusing on hand hygiene practice.

**Methods:** We used a qualitative research methodology to meet the study aim. Combining participant observation (PO) over a seven-month period with semi structured interviews (SSI) to provide an in-depth understanding of activities relating to hygiene and IPC existing on the ward.

**Results:** While most staff and some caregivers, had a good understanding of ideal IPC and understood the importance of good handwashing practices, they faced substantial structural limitations, and scarce resources (both material and human) which made implementation challenging.  For staff, the overwhelming numbers of patients meant the workload was often unmanageable and practicing optimal IPC was challenging.  Caregivers lacked access to basic amenities, including linen and chairs, meaning that it was almost impossible for them to maintain good hand hygiene. Limited access to soap and the erratic water supply for both caregivers and healthcare workers further worsened the situation. Communication challenges between different cadres of staff and with patient caregivers meant that those handling neonates and cleaning the wards were often unaware of outbreaks of drug resistant infection.

**Conclusion: **For IPC to be improved, interventions need to address the chronic shortages of material resources and create an enabling environment for HCWs and patient caregivers.

## Background

Worldwide, the last two decades have seen a radical reduction in under-five mortality ^1^, however neonatal outcomes, especially from neonatal sepsis, have not significantly changed ^2^. Globally, up to a third of all neonatal deaths are attributed to sepsis and between 1990 to 2015, neonatal sepsis had the slowest decline in major causes of child mortality ^3, 4^. Forty-one per cent of under-five deaths were among neonates, of which sepsis accounted for 6% ^5^. In the past three decades, Malawi has made great strides in reducing childhood mortality, meeting the fourth Millennium Development goal in 2013, and reducing it by two-thirds ^6^. However, neonatal sepsis remains a major challenge, evidenced by a recent study conducted in Lilongwe which found it accounted for 23% of neonatal deaths ^7^. Poor outcomes for infants with neonatal sepsis have been worsened by rapid increases in antimicrobial resistance (AMR) in key aetiological agents ^3^.

In low-income contexts, mortality and morbidity from neonatal sepsis are further exacerbated by poor quality care (i.e. paucity of infection diagnostics) and limitations in infection prevention and control (IPC) ^8, 9^. Acquiring a drug-resistant infection can increase the risk of mortality and lead to longer hospital stays, placing an increased economic burden on the already overstretched health services ^10^. The *Klebsiella pneumoniae* pathogen is considered a serious threat to human wellbeing due to a rise in multidrug-resistant strains related to hospital outbreaks ^11^ and has been included on the World Health Organization (WHO) list of priority pathogens for the development of new antibiotics ^12^. In Malawi, *Klebsiella pneumoniae* (KPn) is a major source of neonatal sepsis, and a recent study of the effect of drug-resistant infection established that 45% of individuals with cephalosporin resistant bloodstream infection died, with a hazard ratio for death of 1.44 (CI 1.02-2.04) compared to drug-susceptible infection ^13^. The development of new antibiotics is, however, a slow process, and urgent action to interrupt the transmission of bacteria to vulnerable babies is required. The UNDP ranks Malawi 171 out of 184 on the human development index making it one of the poorest countries in the world ^14^. Access to second and third-line antibiotic therapies is often limited; reducing transmission of drug-resistant infections is vital ^15, 16^.

Most health care-associated infections (HAI), are transmitted via the hands of healthcare workers through either direct contact with patients or through wider environmental contamination, making handwashing a vital preventive strategy ^17^. The WHO has developed universal guidelines on hand hygiene, stressing its importance in the reduction of disease transmission ^18^. However, significant barriers exist to implementing good hand hygiene, particularly in contexts of scarcity. Recent research conducted in sub-Saharan Africa found that suboptimal adherence to hand hygiene practices was shaped by impaired infrastructure, poorly designed facilities, and increased workload ^19^. Lack of awareness and understanding of the mechanisms for pathogen transmission have also been identified as key drivers of inappropriate hand hygiene practices ^20^. In Malawi, two-thirds of health care facilities have piped supplies and one-third have non-piped supplies ^21^. Previous research conducted in clinical settings in Malawi found adherence to hand hygiene to be low ^22, 23^.

As drug-resistant infections become more prevalent, a more in-depth understanding of the factors shaping hand hygiene practices, particularly in low-income contexts, is needed. The study's objective was to understand infection control practices in their social context, focusing on hand hygiene in a neonatal care unit in Blantyre Malawi where there have been frequent outbreaks of *Klebsiella pneumoniae* ^24^. The aim was to develop interventions to reduce the transmission of drug-resistant infections based on evidence generated from the study.

## Methods

### Study site

The research took place in the Chatinkha nursery unit, at Queen Elizabeth Central Hospital (QECH) in Blantyre Malawi. The Chatinkha nursery is a 40-bed referral neonatal unit located within the main hospital grounds. The unit was built in 1980 with significant renovation taking place between 2014–2016. The study ran from September 2018 until March 2020. During the study, the unit was staffed by five qualified nurses, one Clinical Officer and three medical doctors as well as a medical consultant employed by Kamuzu University of Health Sciences. As a teaching hospital, the unit hosts students from medical, clinical, and nursing colleges throughout the Southern region of Malawi. Additionally, the unit provides opportunities for trained medical personnel to gain practical experience and mentorship.

Mothers, and/or female guardians (depending on the circumstance of the mother) are an integral part of delivering patient care in the unit. Key tasks that they support include feeding; changing nappies; ensuring the babies were clean; as well as providing bed linen (and ensuring this was regularly washed). The unit actively encourages babies to receive breast milk (often through a feeding tube). This requires mothers to express breast milk every 2–3 hours, depending on the babies’ medical condition. This meant that mothers and guardians regularly handled their babies. Chatinkha nursery has experienced frequent outbreaks of neonatal sepsis caused by *Klebsiella pneumoniae,* which is why it was selected as the study site ^24– 26^.

### Data collection procedures

This ethnographic study combined participant observation (PO) and semi-structured interviews (SSI) to understand hand hygiene and IPC practices in their social context. The lead researcher (HM) is a nurse-midwife with twenty-eight years of experience working in the Malawian health system and has previously undertaken qualitative research projects.

***Participant observation.*** Between April and September 2019, HM undertook seven months of participant observation. HM was a participant as observer and worked alongside the clinical staff providing essential care to patients during the day and night shifts ^27^. Before the commencement of the study, HM was previously a study coordinator recruiting patients from the unit seeking to understand neonatal sepsis clinical outcomes. This position meant she had established a strong rapport with the core staff as well as a good working knowledge of the unit, including admission processes. All members of staff on the unit were aware of the purpose of HM’s work and no observations were covert. The longer-term engagement and pre-existing relationships allowed her to ask questions and seek clarification from her colleagues during shifts with ease and reduced the likelihood of the Hawthorne effect shaping participants' behaviour enhancing trustworthiness in the data collection process. During all interactions, HM emphasised that she was present to understand practice rather than to judge frontline staff.

During her shifts, HM also spent time with patient guardians and mothers during the patient admission process, during day-to-day care, and whilst providing breastfeeding support. To ensure she spent additional time with patient guardians and mothers, she volunteered to deliver the health talks which were regularly on the ward, and provided guardians with health information. When observing practice, she asked questions sensitively; her care provision during her periods of observation reduced any disruptions in the unit and enhanced the relationship between HM and the participants. She took brief field notes during her shifts, expanding on them during breaks, and completed them following her shifts. Insights from the participant observation were then used to inform the guide for the semi-structured interviews. We did not use a structured data capture tool for the observations, as we wanted to capture the wider social factors shaping the behaviour of staff.

***Semi-structured interviews.*** All interviews were conducted by HM in a mixture of Chichewa and English for frontline workers, and Chichewa with guardians. We purposively sampled both frontline staff (n=13) and caregivers (n=10) for SSIs. The thirteen frontline staff included medical, nursing and ancillary staff. Included in these interviews were 1 medical doctor, 1 clinical officer, 1 student clinical officer, 1 cleaner, 1 patient attendant, 3 student nurse/midwife technicians, 4 nurse/midwife technicians and 1 state registered nurse. We included ancillary staff, to reflect the range of cadres engaged in infection prevention practices, which included hand hygiene. The ten caregivers were either mothers or guardians of babies who were admitted to the ward. We sought to ensure a range of experiences of caregivers was represented, by sampling those whose babies had recently been admitted, and those who had babies that had been on the ward for longer than one week. All interviews were held in a private office in the unit, allowing the staff and guardians to be close to the unit while minimizing any disruption to the care practices on the ward. The length of the interview was between 45–60 minutes. Separate topic guides were developed for frontline workers and mothers/guardians interview. For frontline workers, key topics explored were: knowledge of infection sources, hand hygiene, potential challenges faced in implementing IPC and what measures can be put in place to address these challenges. During interviews with mothers and guardians, topics explored included: knowledge and understanding of infection and IPC, handwashing practices and the barriers and enablers to implementing good practice. A copy of the interview guide can be found in the Extended data. All interviews were taped, and the recordings were downloaded on a secure laptop translated into English and transcribed by HM.

***Data analysis.*** To analyse the data, we drew on the framework approach ^27^. Analysis began from the first week of data collection, with HM and EM holding weekly debriefing sessions during which they identified themes, any unexpected findings, and any new avenues to explore. Only EM and HM had access to the transcripts and the patient information. All data (including fieldnotes and transcripts) were imported into NVIVO 12 (for working with qualitative data an alternative could be open code) and coded using a thematic approach. HM and EM developed the initial coding frame together. This was based on viewing the transcripts and field notes. Once the initial coding frame had been developed and theme summaries had been developed, they were presented to the wider group of researchers. Following discussions, it was then updated by HM and a chart was developed to support interpretation. During the study, HM held regular debriefing sessions with ward managers, cleaning service managers, qualified nurses, and patient guardians to share findings and seek their reflections. At the end of the study, she presented findings to the hospital Department of Paediatrics and the Chatinkha Neonatal Unit. This ongoing engagement allowed for regular participant checking and discussion of implications for practice.

### Ethical considerations

Ethical approval was obtained from College of Medicine Research Ethics Committee (COMREC P. 08/18/2460) and the Liverpool School of Tropical Medicine Research Ethics Committee (Ref 17-083). Informed written consent was obtained from all health workers before interviews and observations began. All guardians and mothers provided informed written consent for interviews. Due to the high flow of patients, verbal consent for observations was obtained from mothers and guardians. The mortality rate on the ward was high and conditions particularly for mothers who had recently given birth were challenging. HM and EM’s regular debriefing sessions also explored the ethical challenges HM faced during the data collection. Meetings also provided HM with an opportunity to discuss some of the more upsetting experiences witnessed during the shifts, particularly when a mother had lost their baby.

## Results

Overall, we found there were significant gaps between ideal and actual hygiene and infection control practices, including handwashing and what frontline workers and caregivers were able to enact. We structure the findings around two key themes. The first theme explores how structural and health systems barriers shape IPC focusing on how the provision of key materials including water, sanitation, and hygiene (WASH) facilities, and working conditions for staff and caregivers. The second theme explores individual barriers to enacting ideal practice which relates to the knowledge of frontline workers and caregivers. In this theme, we demonstrate how knowledge is shaped by training and communication practices on the ward.

### Structural and health systems issues shaping infection control practices

***Water, sanitation, and hygiene (WASH) facilities for the ward.*** There were three handwashing points available inside the ward, with one further handwashing station at the entrance to the ward. There were large water storage buckets in the kitchen and the sluice area. Water stored in the buckets was primarily used for cleaning surfaces and floors of the ward. Spray bottles with methylated spirits were used to sterilise equipment such as thermometers and stethoscopes. These were found on the ward but were frequently empty. Water shortage was a substantial challenge. The taps ran dry on approximately three days every week and there was no backup supply. The water cuts usually lasted approximately five hours, but the erratic nature of the shortages left staff and caregivers unable to predict when water would, or would not, be present. Soap was frequently absent from handwashing facilities during the study period. When there was no water, HCWs improvised and used saline drips or sprayed the methylated spirit intended for clinical use onto their hands.

As can be noted in the quotes below, neither were seen as ideal practice for HCWs:

“ *We have resorted to using normal saline infusion drips. We open and use them for hand hygiene together with the methylated spirit.” [SSI, student male nurse]*
  “ *We use methylated spirit to wash our hands. Not rubbing but using it instead of water, but if we do that, our hands become so dry and hard. Most people don’t like doing that, the hands become rough and [it] doesn’t feel good.*” *[SSI, male clinical officer]*

Infrequently there was hand sanitizer provided on the ward to staff. HCWs often complained about the quality of the product, which left residue on their hands. Some staff, predominantly doctors and medical students, did carry hand sanitisers, which they would use on their own hands. This was markedly different for nurses who were rarely observed with their own individual hand sanitiser and may reflect the different economic positions of the two groups. There were few options for the HCWs to dry their hands. HCWs used rolls of gauze swabs left on the nurses’ station, or some staff used their handkerchiefs, which they stored in their pockets. Despite the intermittent availability of soap and water to facilitate hand hygiene, provision of other protective wear such as aprons and gloves was found to be in adequate supply.

*“We just work without soap. If it’s not there, then there is nothing we can do. We go on working without soap for handwashing or cleaning. But for the gloves, it’s not likely that they run out of stock. Even aprons are always available, mainly its soap and chlorine that is usually in short supply.” [SSI female Nurse Midwife]*

***WASH opportunities and barriers for caregivers.*** While caregivers operated within the same environment as the HCWs, they faced additional challenges in washing their hands. On the ward, only HCWs and students were permitted to use hand-washing points. If a mother or guardian tried to use the handwashing facilities or the hand sanitiser on the ward, hospital staff would reprimand them and redirect them to the washing station situated outside the ward. When the spray bottles of methylated spirits were full the caregivers were not permitted to use them. This is likely to have contributed to the observed inconsistent and low level of hand hygiene among the guardians and mothers.

Mothers and guardians of sick babies were accommodated in the nearby postnatal ward and spent most of the time on the ward sitting on the floor. They were expected to visit their babies every 2–3 hours around the clock to perform a critical role in providing care for the babies on the ward. Only in exceptional circumstances, such as the mother dying, would nurses feed or change babies, all the care fell on the mothers or guardians, meaning they handled the babies frequently. The caregivers faced significant barriers in enacting good hygiene and infection control practices. As can be seen from the fieldnote, the lack of chairs and limited access to hand-washing facilities is likely to have also contributed to infections spreading within the unit:

*It’s the first day of the week and the ward is congested with weekend admissions. Over 20 mothers are sitting on the floor because the chairs in the unit are not enough to accommodate everybody. I watch as one of the mothers sit on the floor expressing milk into a feeding cup, carefully measuring the amount. The baby is on oxygen, and therefore requires feeding through a nasal gastric tube. The mother stands up, using a syringe she sucks up the milk and then connects the syringe to the feeding tube. Her hands and the feeding cup have been on the floor due to the lack of space. It makes me reflect on how challenging it is for mothers to perform good hand hygiene.* [Field note May, 2019, HM]

***Overwhelming staff workload and challenging working conditions.*** Understaffing was seen by all the healthcare staff as a key challenge. This was particularly pronounced during the night and weekend shifts when staff numbers were reduced.

*“…as I mentioned before that in the past, we used to have few patients. We could consider the ward to be full when we had 20 patients. But now we are having a lot of patients with few nurses, we have 3 nurses on night duty to look after 50–70 babies, with new admissions still coming in…”* [SSI, female Nurse/Midwife]

The unit was severely understaffed. During the study period we found an average of four nurses during the day and three to cover the night shift. Night shifts were more challenging because there were fewer auxiliary staff such as patient attendants and student nurses, placing a higher burden on the staff. There was a noticeable difference between the day and night shifts, with less frequent handwashing happening during the night than during the day. Frontline healthcare workers frequently felt stressed and overwhelmed by the workload they faced. Healthcare workers had a good understanding of “ideal” IPC but often felt that the workload hindered them from implementing this. As it can be seen in the quote, where capitalisation denotes the interviewee raising their voice, health workers felt anger and concern at the situation:

*“We are supposed to wash our hands with soap or use a spirit hand rub before and after handling a baby. We are also supposed to clean any cot that a baby has been removed from. When conducting any clinical procedures, we must wash our hands, put on gloves and apron and we have to follow sterile techniques. But sometimes maybe because of the pressure of work…. it happens that, maybe one baby becomes critically ill and requires urgent attention, we just transfer this baby to another place without considering whether it’s clean or not. Our main aim is to save the life of the baby without considering whether the area is clean or not. OUR INTENTION IS JUST TO SAVE THE BABY’S LIFE, RIGHT? without considering whether the cot is clean or not we don’t even know what happened to that cot before.”* [SSI, female Nurse/Midwife]

In the quote, the nurse stresses the importance of dealing first with life threatening situations. In a neonatal referral unit, babies often came into the unit in a critical state and staff described responding in a crisis mode, prioritising critical care above all other activities. As can be seen from the fieldnotes below, the staff were dealing with extremely sick babies in a fragile health system. For mothers, their experiences of trying to navigate the system could be extremely challenging.

*I am working the night shift, there is two other nurses. The ward has become quiet after being busy with a few additions from the labour ward. It’s 3 am a young lady walks in with her baby in her arms. She is extremely distressed. She gave birth at a health care facility about 5km away a few hours ago. The baby developed breathing difficulties after birth, so the facility staff called an ambulance. The ambulance dropped the lady and her guardian at the gate of the Central Hospital. The hospital is a sprawling set of buildings, and Chatinkha is situated at the opposite end of the hospital making the walk, after giving birth long and slow. By the time the lady located the unit, her baby has stopped breathing. She handed over a silent baby, we tried to resuscitate but it was not possible. I went to check on her and find out how she was getting home. Mothers want to take their babies home if they have passed, the minibuses [the transport most people use] won’t transport mothers in this situation. She explained she was going to walk her. She wrapped her baby into a piece of chitenje and set off. As I left the hospital that morning, I could not move on from the overwhelming feeling of hopelessness and sadness for the mothers who must endure so much.* [HM fieldnotes May 2019]

There were times when the government paid for external locum staff and newly qualified students to support the permanent staff. Having other nurses coming to the unit as locums eased some of the staff shortages and were believed to improve overall care. As noted in the quote below, the nurse articulated the importance of having additional support.

*“To have more nurses on a shift helps a lot, we share responsibility well and the workload is lessened. …. When we have few nurses on duty, it becomes very difficult to do everything that we have to do as nurses. So, what happens is that we just concentrate on the clinical care of the babies, such as giving medication and may be resuscitating babies who may need it. In that way, we can’t consider cleaning as a priority, even changing the water in the suction bottles. We forget to do all that because we have the pressure of work, and we are few nurses on the shift. But when we are more nurses on duty, I have noted that things go very well*.” [SSI, female Nurse Midwife]

However, there were times when locum nurses were not always provided with a sufficient orientation to the procedures on the ward. This meant that there were observable differences in the interactions between locum staff and permanent staff particularly when communicating key aspects of IPC and ward procedures to mothers and guardians. Nursing students, were not always provided with sufficient supervision, which at times left the nurses concerned about how they were providing care and performing infection control practices.

***Limited cots and overcrowded wards.*** The number of cots available ranged between 40–50 with an average of 45. The unit typically ran at an occupancy rate of between 30–75 babies, with an average of 53 babies on the unit at any time. During the study, the unit admitted between 5 and 18 neonates per day. Admissions were made 24-hours a day. The babies were referred from Queen Elizabeth Central Hospital labour ward, from health centres around Blantyre, as well as from District Hospitals in the Southern Region. The babies were admitted for various reasons ranging from prematurity, infections and congenital defects which required surgical interventions. The limited number of cots and the high number of admissions meant that it was often challenging for the clinical team to implement good IPC, predisposing the babies to infections, including those that were drug-resistant. During the interviews, the clinical team often voiced frustration that these were the conditions they were working within noting that things were worsening over time. The clinical staff had a clear understanding of the risks of cot sharing but the limited resources meant they were unable to change the situation.

*“This started some few years ago because of lack of space. In the past, we could consider the ward to be full when we had 17 to 20 babies. But now when we say the ward is full, we have 50 to 70 babies, and the space is so limited. It's another point that concerns me, we put four babies on one Resuscitaire, and we are not aware of who may have an infection. We sometimes put a baby new to the ward, next to those who have been on the ward for longer. This is a burden*.” [SSI Midwife]

Caregivers also spoke about the ways this could drive infection, but acknowledged that the clinical team had little choice:

“ *I believe that in some cases it’s because we do not have a choice but, you’d see two or more babies sharing a bed. The two babies may have different cases, but since they are being kept in the same place, it is very easy for them to share infections with each other. If there was a way that every baby should be put on their own place, that would prevent them from sharing infections to one another.*” [SSI, female Guardian]

***Restricted use of hospital linen.*** During the interviews, participants reflected on the provision of hospital linen and how this had changed over time. In the past hospital, linen was provided in the cots. However, due to financial shortages in the hospital, the linen service had been discontinued. Women had to provide linen for their babies. They often used small porous pieces of cloth locally known as *chitenje*, both to serve as nappies and wrappers to keep the babies warm. The hygiene status of *chitenjes* was uncertain as mothers and guardians had to wash and dry them in the hospital. The drying often took place on a grassy space outside the ward and at times without soap if the family could not afford to provide it.

*“Some time ago, the babies were provided with hospital linen. This linen was being washed and dried here in the hospital. Nowadays mothers use their linen from home. We are not even sure how these mothers care for the chitenjes. I just observe that they dry them on the grass outside.”* [SSI, female Nurse Midwife]

### Knowledge and communication shaping infection control practices

***Knowledge of infection prevention and control practice.*** We found that HCWs had a good understanding of IPC, with their knowledge coming from their clinical training. The neonatal care unit previously held ward meetings about IPC and hygiene promotion, and these meetings served as a source of information-sharing for the ward staff. The staff felt the meetings required participation from everyone working in the ward, seeing it as imperative for the effective implementation and continuation of hygiene promotion. However, some staff, including cleaners and hospital attendants, were rarely, if ever, invited to ward meetings, meaning this group of staff missed information and training opportunities; as a result, they did not feel empowered to contribute to IPC. When asked about this, one female hospital attendant shared the following:

*“We are not included in the meetings. I can’t remember when we last had a meeting together.”* [informal conversation with female patient attendant]

***Lack of trainings and health talks on infection control practices.*** During discussions, both in interviews and informal conversations, HCWs felt they lacked opportunities for training to ensure they were up to date on best practices on hygiene promotion and infection control.

“ *I have never attended a single seminar on infection control since I started working in this unit*.” [SSI, male Nurse Midwife]

HCWs felt that training would also create space for reflective feedback. There was a consensus among the HCWs on the need to have such training among all cadres, to address common challenges and share current information on infection prevention and hygiene promotion.

*“[I] would be happy to get additional information on that because we don’t want the infections to be spreading. We know there are a lot of barriers to reducing the burden of infection in our context, but we have to stop it from spreading. So, if there is any new information which may help in reducing infection transmission, will be happy to have that*.” [SSI, male clinician]

***Knowledge and management of drug-resistant infections.*** HCW had a good understanding of drug resistant infections and how this might impact treatment outcomes for the babies. Doctors and medical students had a more in-depth understanding of drug-resistant infection in comparison to nurses. This can be seen in the quote below:

*“The resistance that comes in one’s body against the medication that is given to cure some pathogens in the body. This resistance makes the pathogens be in the body and continue multiply and cause illness in the body.”* [SSI, male clinician]

When babies were diagnosed with a drug-resistant infection, doctors could access the laboratory results on their mobile phones, and then request that the nurses place the babies in isolation. However, the nurses were not always informed of the diagnosis. This meant that nurses were not aware of the need to use personal protective equipment or to increase hand-hygiene practices to reduce the spread of the pathogen within the unit. Furthermore, babies were not screened for carriage of AMR bacteria and were only moved from the main ward once a drug resistant infection had been confirmed, which meant they could spend up to seven days in the main ward, which could contribute to the transmission of drug resistant infection to other babies.

***Communication and information sharing with caregivers.*** During the admission process, nurses were supposed to give a briefing to caregivers regarding best hygiene practice when handling the babies as well as clear guidance on how to follow the IPC procedure on the ward. However, HM found gaps with this in practice. Firstly, during busy shifts nurses were only able to spend a limited period with the caregivers due to the often-heavy workloads. The caregivers at times were given very limited information about the babies’ condition and the ward's practices and procedures regarding infection control. Secondly, only one caregiver was allowed to facilitate the admission process, yet multiple caregivers may be involved in providing care for the babies, particularly if the mother was unwell or required rest (having recently given birth). Consequently, not all caregivers received the appropriate information and advice to follow. However, during the interviews with caregivers, some but not all, demonstrated a good understanding of the importance of practising good hand-hygiene to prevent the spread of infection. Those who were caring for babies who had been on the ward longer than one week had a better understanding of the importance of hand washing.

*“If we don’t wash our hands before and after caring for the baby, we can put the baby at risk.”* [SSI guardian]

During the interviews, the caregivers also reflected on HCWs hand hygiene practices and the ways this may shape infection:

*“Well, judging on the incidents here, when a baby is put on oxygen, and they so happen that the baby has removed the prongs. We call the healthcare workers around, some clean their hands before attending to the baby while others just attend to the babies without doing that because they are in a hurry, I don't think that's healthy for the baby but then again, most women really don't mind as long as their baby has been helped.”* [SSI guardian]

As can be seen from the interview, the first concern for the caregivers was to ensure that their babies received medical attention.

## Discussion

This ethnographic study was conducted to understand IPC practices, focussing on hand hygiene in a neonatal referral unit in Blantyre, Malawi following a series of outbreaks of neonatal sepsis associated with antimicrobial resistant *K. pneumoniae.* In the study, we sought to understand how individual knowledge and the broader structural and health systems factors shaped IPC, particularly hand hygiene practice. By combining participant observation with semi-structured interviews, we were able to capture data on both reported and observed behaviour. Building on HMs relationships in the unit and her previous knowledge procedures allowed for an in-depth exploration. We found HCWs and some caregivers had a good understanding of the importance of implementing ideal hygiene practices but faced daunting structural limitations and scarce resources (both material and human) which significantly impacted practice. The overwhelming workload of HCWs, particularly during the night, meant that staff often failed to enact good hand hygiene or IPC practices. When the soap was absent, there was scarce hand sanitisers and erratic water provision they simply had to “make do” with the materials they were able to access. The chronic shortage of cots meant sharing was common and containment in the event of a disease outbreak challenging. Power hierarchies shaped IPC practice for frontline staff and caregivers. If a baby was diagnosed with a drug-resistant infection, the information was rarely cascaded to other staff involved in providing care beyond the doctors. Cleaners and patient attendants were rarely included in ward meetings or training on IPC. Caregivers were policed by hospital staff if they did try to use the handwashing basins or hand sanitiser on the ward.

Our work suggests a critical need to address WASH infrastructure limitations within the ward and improve hand hygiene access for guardians. This reflects findings from other studies that emphasise the need to support and enable hand hygiene among HCWs and all those involved in clinical care ^28– 30^. A Cochrane review of interventions to improve hand hygiene compliance in patient care found that a multimodal package of interventions including alcohol-based hand rubs, education, reminders, performance feedback and managerial support is applicable to all settings ^31^. In water-constrained environments, such as Malawi, alcohol-based hand rubs are likely to be an important intervention. In Tanzania, research demonstrated that alcohol-based hand sanitizer was an acceptable means of hand hygiene ^32^. However, our research also found that the quality of the hand sanitizer impacted use, with staff complaining about cheaper formulas leaving their hands feeling sticky.

Little research has been conducted to date on guardians’ knowledge and practices regarding hand hygiene, especially in low-income contexts where they are essential to patient care ^33^. However, one report in Malawi showed guardians felt their practice was improved when information was shared with them by trained health personnel ^34^. Gaps in sharing information with parents and guardians present an infection control risk. There is therefore an important need to ensure better communication on IPC with parents and guardians, in a way that is context-appropriate and supportive.

Structural violence is a concept made popular in medical anthropology and wider global health research by Paul Farmer ^35^. The analytical concept brings to the fore the often hidden ways that structures of inequality such as poverty, racism and discrimination, negatively impact the lives and well-being of affected populations ^35^. If we apply the concept of structural violence to the outbreaks of *K. pneumoniae* in the Chatinkha nursery we can see the ways in which lack of (human and financial) resources drive infection and death, creating extreme health inequalities. In the absence of water, soap, and sufficient staff to provide care to all those admitted we can see the inevitability of infections spreading. The absence of these resources is driven by social, economic, and political configurations that mean Malawi is one of the poorest countries in the world. Caregivers, HCWs and babies experience harm working and caring in these extremely difficult circumstances which they have little power to change. Without urgent interventions to alter the structural factors and increase material resources, drug-resistant infections are likely to lead to higher rates of mortality creating more harm to caregivers and HCWs.

### Implications for clinical practice

Our paper renders visible the extremely challenging conditions that HCWs and caregivers face in the Chatinkha unit. While focusing on IPC and hand hygiene practice we can see how conditions in the unit shaped practice. Interventions to improve IPC practices need to be introduced in a supportive and inclusive way, acknowledging these barriers. The need for improved WASH facilities and a stable water supply is clearly demonstrated. The introduction of high-quality hand sanitisers is likely to be a useful intervention. Addressing power dynamics, including ensuring the equitable provision of the resources available and improving communication with all those providing patient care, including mothers and guardians could be an important intervention. Ensuring that training on IPC is open to all members of staff including cleaners, caregivers, and patient attendants could help improve knowledge and communication. When babies are suspected of having a drug-resistant infection, all staff should be alerted.

### Limitations of the study

The study was situated on a busy neonatal intensive care unit, which could have up to 70 babies admitted at one time. Data was collected by one individual, which means it was not possible to observe all aspects of care. HM also had pre-established relationships with staff, which may have shaped their interactions. However, the long-term nature of the study meant HM observed staff over a longer period emphasising that this was not a study about people being “right or wrong” but rather understanding IPC and hand hygiene in the context it was occurring.

## Conclusion

Drug resistant infections are increasing rapidly across the world. The structural and material conditions of low-income countries mean that the ramifications will be more acutely felt in these settings. Our work speaks to the critical need to provide improved WASH infrastructure, address staff shortages and cocreate solutions that meet the hygiene and IPC challenges that staff and caregivers encounter.

## Data Availability

Data remain the property of the Malawi government, as per Malawi legislation. It is not possible to fully anonymise the data as the transcripts and fieldnotes contain highly sensitive and personal narratives from infants receiving care in a specialised neonatal intensive care unit. Researchers wishing to access the fieldnotes and transcripts should write to the principal investigator ( emacpherson@mlw.mw) with a detailed description of the purpose for requesting the transcripts and fieldnotes. Requests for fieldnotes will be evaluated by the MLW research strategy committee in accordance with the MLW data department SOP. Individuals provided with data, will be requested to sign a confidentiality agreement outlining the conditions and purposes data can and cannot be used, and procedures for preserving anonymity. OSF: A qualitative study exploring health workers and patient caregivers’ hand hygiene practices in a neonatal unit in Blantyre, Malawi, implications for controlling outbreaks of drug-resistant infections. DOI: https://osf.io/5qsna/ ^31^ This project contains the following extended data: Topic guide Front line_Chichewa.pdf Topic guide Front line_Eng.pdf Topic guide Guardians_Chichewa.pdf Topic guide Guardians_Eng.pdf Data are available under the terms of the Creative Commons Attribution 4.0 International license (CC-BY 4.0).
