# Supplementary material for: A qualitative study exploring hand hygiene practices in a neonatal unit in Blantyre, Malawi: implications for controlling healthcare-associated infections
Source: Wellcome Open Res. 2023 Apr 21;7:146. Originally published 2022 May 6. [Version 3] doi: 10.12688/wellcomeopenres.17793.3 (PMC10170178; doi:10.12688/wellcomeopenres.17793.3)
Supplement: Peer review history

# Peer review: Reviewer response for version 3

**DOI:** 10.21956/wellcomeopenres.21448.r56434

**Published:** 2023-05-09

**Authors:** Maggie Zgambo

**Affiliations:** 1 School of Nursing and Midwifery, Edith Cowan University, Joondalup, Australia

No further comments to make. Best wishes.

Is the work clearly and accurately presented and does it cite the current literature?

Partly

If applicable, is the statistical analysis and its interpretation appropriate?

Not applicable

Are all the source data underlying the results available to ensure full reproducibility?

No

Is the study design appropriate and is the work technically sound?

Yes

Are the conclusions drawn adequately supported by the results?

Partly

Are sufficient details of methods and analysis provided to allow replication by others?

No

Reviewer Expertise:

NA

I confirm that I have read this submission and believe that I have an appropriate level of expertise to confirm that it is of an acceptable scientific standard.

# Peer review: Reviewer response for version 2

**DOI:** 10.21956/wellcomeopenres.21154.r55281

**Published:** 2023-03-24

**Authors:** Maggie Zgambo

**Affiliations:** 1 School of Nursing and Midwifery, Edith Cowan University, Joondalup, Australia

I have reviewed this article and I am happy with the improvements made to the manuscript. I do not have any significant issues to raise at this moment. However, before I recommend accepting the article for indexing, please may you address the following concerns ***.***

 The team for this manuscript has done a wonderful job to improve their work. Great improvement is seen in all sections. I hope you will find a few issues below helpful. Best wishes. 

**Abstract**

- Please add the methodology used i.e ethnography (see also comment below on methodology)
- Please delete PO, SSI and note the use of HCWs
- Add details of participant and sample size to the methods section
- The background can be shortened
- Comment on data analysis
- Instead of randomly presenting findings, please state the identified themes and include relevant information under each theme, briefly

**Body**

- There are just a few minor grammatical errors to correct eg Under restricted use of hospital linen...it should read ‘In the past, hospital lines…?”
- There are several types of ethnographic studies. Could the authors include the type of ethnography used in this study? I am assuming it is focused ethnography. Also, please add 2 or 3 sentences to justify the choice of this method. 
- Great improvement is seen under data analysis section. However, it is still not clear and lacks more details for reproducibility. Did the authors follow any thematic analysis framework to analyse data? How was coding done? How did categories and themes emerge? Etc. Further to this, and as asked last time, how was data from observation analysed and merged to the SSIs data?
- Some quotations can be shortened to include what is necessary and related to the main narration. Eg field notes starting with " *I am working the night shift, there is two other nurses. The ward has become quiet after being busy with a few additions from the labour ward. I...'*

Is the work clearly and accurately presented and does it cite the current literature?

Partly

If applicable, is the statistical analysis and its interpretation appropriate?

Not applicable

Are all the source data underlying the results available to ensure full reproducibility?

No

Is the study design appropriate and is the work technically sound?

Yes

Are the conclusions drawn adequately supported by the results?

Partly

Are sufficient details of methods and analysis provided to allow replication by others?

No

Reviewer Expertise:

NA

I confirm that I have read this submission and believe that I have an appropriate level of expertise to confirm that it is of an acceptable scientific standard, however I have significant reservations, as outlined above.

# Peer review: Reviewer response for version 1

**DOI:** 10.21956/wellcomeopenres.19694.r51718

**Published:** 2022-08-22

**Authors:** Maggie Zgambo

**Affiliations:** 1 School of Nursing and Midwifery, Edith Cowan University, Joondalup, Australia

In general, this is an interesting topic and I commend the authors for taking an interest in this area of study. However, some issues need rectifying throughout the document. The paper might also benefit from careful proofreading and editing to improve readability and cohesion.  

 Best wishes.

 Title :

 The title is a bit wordy- consider trenching some word

 Introduction 

1. Is this in Malawi? ‘From 1990 to 2015, neonatal sepsis had the slowest decline among the major causes of child mortality”
2. Would be lovely to include statistics on the reduction of neonatal mortality in Malawi
3. In the last sentence of the first paragraph, you mention that neonatal outcomes from sepsis have not changed significantly, and you emphasise the worsening of poor outcomes following neonatal sepsis in the next sentence. Please review and revise for clarity
4. Introduce the acronym -WHO-
5. A good discussion on drug-resistant infection is given in the introduction section, however, little is presented on hand hygiene, which is the focus of the paper. What is the current status of hand hygiene in Malawi? Are there any available programmes promoting hand hygiene in hospitals? Also, would the authors include epidemiological information to highlight the significance of the problem (drug resistance or Klebsiella pneumoniae) in Malawi?
6. Could the authors reword the ‘purpose’ of this study ( *The purpose of this research was to inform the development of interventions to reduce the transmission of drug-resistant infections*)? I am assuming the sentence is meant to justify the aim of the study. As it stands, it reads like there are two different aims for this study. 

Methods

 Study site

1. Kindly relocate this information as it is not directly linked to Chatinkha ‘The UNDP ranks Malawi 171 out of 184 on the human development index making it one of the poorest countries in the world.’
2. I am wondering how many students were in the unit during data collection. Also, QUECH is a teaching hospital - that’s why it has students (not because of being a referral hospital)
3. What measures were put in place to minimise HM’s influence on selecting colleagues to participate and influencing responses from both colleagues and caregivers in the study?
4. How and when were these observations carried out (did HM work alongside other workers because of the study or during her shifts?)? Was everyone in this unit observed? What type of observations were these? Any tools used to observe participants? What was being observed? How were data recorded from these observations? What research skills did HM have before undertaking observations? Were health workers the only participants that were observed?
5. Did you mean disturbance/disruption in this sentence ‘while minimizing any ***distribution*** to the care practices on the ward’?
6. How many nurses and which cadres within ‘the medical staff’ were included in this study?
7. What language did you use during interviews of both health workers and caregivers? Were there any translations done? How was this undertaken? Who interviewed the participants? How was the data corrected? Did they use different interview guides for participants?
8. You need a heading for data analysis.
9. Data analysis procedures are not explained sufficiently or clearly. Please highlight procedures undertaken during data analysis ie how was the coding done? Did you follow any guidelines? Who did the coding? How were themes identified? Were there any disagreements? How was this resolved? Etc…
10. Please reorganise information on data analysis for cohesion. Information on tape-recording should move to data collection, and steps undertaken should follow through clearly in a chronological manner.
11. Considering that only HM and EM had access to data, I am wondering how the ‘wider’ group agree/adopt the ‘developed themes’ without access to data.
12. Also, I am not sure how presenting the findings to the unit and department validated your data
13. A coding tree is needed

Ethical consideration

1. Could you please relocate this information as it is not discussing how you met ethical requirements for the study ‘The mortality rate on the ward was high and conditions particularly for mothers who had recently given birth were challenging? HM and EM’s regular debriefing sessions also explored the ethical challenges HM faced during the data collection. Meet-ings also provided HM with an opportunity to discuss some of the more upsetting experiences witnessed during the shifts, particularly when a mother had lost their baby.’ 
2. How was the verbal consent obtained from parents and guardians for observations? Explaining further how these observations were carried out for this study group would be great.
3. You might want to omit the name of the unit for confidentiality.

General

1. How was trustworthiness (components) achieved in this study?
2. Please utilise the Coreq checklist for reporting qualitative studies https://www.equator-network.org/reporting-guidelines/coreq/
3. How did you merge data from field notes, observations, and interviews in your data interpretation?

Results & discussion

1. Justifying actions should come under the discussion section e.g. cost to purchase personal hand sanitiser
2. The authors state that the unit needed 20 nurses, is this claim based on any national staffing guidelines?
3. Present demographic data of participants as well
4. I suggest using data from participants who consented to the study only, data collected during interviews/obs/field noted. Data from informal conversations should be excluded.
5. Choose one format for quoted references
6. Were there any positives identified in this study considering that you aim at identifying practices? 
7. The discussion has not adequately demonstrated an engagement with extant literature on the subject. Also, implications are not suggestive of the way forward regarding practice, research or policies. The so what or what next, and who should do it are not coming out clearly.

Is the work clearly and accurately presented and does it cite the current literature?

Partly

If applicable, is the statistical analysis and its interpretation appropriate?

Not applicable

Are all the source data underlying the results available to ensure full reproducibility?

No

Is the study design appropriate and is the work technically sound?

Yes

Are the conclusions drawn adequately supported by the results?

Partly

Are sufficient details of methods and analysis provided to allow replication by others?

No

Reviewer Expertise:

Pediatric Nursing, Health Promotion, Health risks, Qualitative studies

I confirm that I have read this submission and believe that I have an appropriate level of expertise to confirm that it is of an acceptable scientific standard, however I have significant reservations, as outlined above.

# Peer review: Reviewer response for version 1

**DOI:** 10.21956/wellcomeopenres.19694.r50340

**Published:** 2022-05-31

**Authors:** Maggie Montgomery

**Affiliations:** 1 Water, Sanitation, Hygiene and Health Unit, World Health Organization, Geneva, Switzerland

The qualitative study exploring health worker and patient caregivers' provides important insights into a subject that has not received sufficient inquiry, especially considering that maternal and newborn infections remain high and many of these can be prevented. I commend the authors for taking on this important piece of work.

 I especially appreciated the nuanced observations about the power dynamics of who has access to hand hygiene supplies with doctors and medical students having priority. It would be useful to know if the study inquired/discussed with doctors/medical students their observations and the reactions from these staff. It seems any solution and more equitable provision of supplies, requires engagement with those controlling the resources.

 Also, while I realize this is a qualitative study, it would be interesting to graphically display frequency of responses to better and more rapidly/visually display inequities in access to hand hygiene supplies and frequency of hand hygiene practices by the key groups (e.g doctors, nurses, caregivers, cleaners).  Also, there seems to be differences in the quality/type of hand sanitizer, which again is an important insight and not discussed often in the literature. Could the authors speak a bit more on this-is it due to resources (e.g. doctors given better quality sanitizer or having the resources to buy higher quality?) Finally, it might be useful to compare the national figures on WASH access in health care facilities in Malawi to the study hospital.

Is the work clearly and accurately presented and does it cite the current literature?

Yes

If applicable, is the statistical analysis and its interpretation appropriate?

Yes

Are all the source data underlying the results available to ensure full reproducibility?

Yes

Is the study design appropriate and is the work technically sound?

Yes

Are the conclusions drawn adequately supported by the results?

Yes

Are sufficient details of methods and analysis provided to allow replication by others?

Yes

Reviewer Expertise:

Environmental engineering, water quality, water, sanitation and hygiene in health care facilites.

I confirm that I have read this submission and believe that I have an appropriate level of expertise to confirm that it is of an acceptable scientific standard.
